# Changes in health in Belgium, 1990–2016: a benchmarking analysis based on the global burden of disease 2016 study

**DOI:** 10.1186/s12889-018-5708-y

**Published:** 2018-06-20

**Authors:** C. Maertens de Noordhout, H. Van Oyen, N. Speybroeck, B. Devleesschauwer

**Affiliations:** 1 Institute of Health and Society (IRSS), Université catholique de Louvain, Clos Chapelle-aux-Champs, 30 bte B1.30.15, 1200 Brussels, Belgium; 2 Department of Epidemiology and Public Health, Sciensano, Brussels, Belgium; 3 Department of Public Health, Faculty of Medicine and Health Sciences, Ghent University, Ghent, Belgium; 4 Department of Veterinary Public Health and Food Safety, Faculty of Veterinary Medicine, Ghent University, Merelbeke, Belgium

**Keywords:** Belgium, Burden, Disease, Benchmarking

## Abstract

**Background:**

Despite increasing of the Belgian health expenditures, several indicators related to population health showed poor results. The objectives of this study were to perform an in-depth analysis of the secular trend of Belgian health status using the Global Burden of Disease (GBD) 2016 study results for Belgium, and to compare these results with other European countries.

**Methods:**

We collected results of the Global Burden of Disease 2016 study through the GBD results and visualization tools. We benchmarked Belgian GBD results with the other initial members of the European Union (EU15).

**Results:**

Belgium performed significantly better in 2016 than in 1990 in terms of age-standardized (AS) Year of Life Lost (YLL) rates but not significantly different in terms of AS Year Lived with Disability (YLD) and Disability-Adjusted Life Year (DALY) rates. The contribution of AS YLDs to total of AS DALYs increased from 1990 (42%) to 2016 (54%). Although AS YLD and DALY rates did not seem to differ between Belgium and the EU15 from 1990 to 2016, the ranking of Belgium among the EU15 in terms of AS DALY and YLL rates was worse in 2016 than in 1990. Belgium had significantly higher AS YLL rates for lower respiratory infections (B: 264 AS YLLs [95% uncertainty interval [UI] 231–301] per 100,000; EU15: 188 AS YLLs [95%UI 168–212] per 100,000), chronic obstructive pulmonary disease (B: 368 AS YLLs [95%UI 331–407] per 100,000; EU15: 285 AS YLLs [95%UI 258–316] per 100,000) and tracheal, bronchus, and lung cancer (B: 785 AS YLLs [95%UI 699–879] per 100,000; EU15: 613 AS YLLs [95%UI 556–674] per 100,000).

**Conclusion:**

Belgium’s ranking among the EU15 in terms of AS YLL and DALY rates decreased from 1990 to 2016. Significant health gains appear possible by acting on risk factors directly linked to a significant part of the Belgian burden of diseases, i.e., alcohol and tobacco consumption, and high body mass index. National burden of disease estimates can help defining Belgian health targets and are necessary as external validity of GBD results is not always guaranteed.

**Electronic supplementary material:**

The online version of this article (10.1186/s12889-018-5708-y) contains supplementary material, which is available to authorized users.

## Background

The Belgian health system is characterized by a compulsory national health insurance which covers almost the entire population, a free choice of physician, and predominantly fee-for-service payment. The system aims to ensure solidarity between rich and poor, and between healthy and sick people. The mandatory health insurance covers more than 8000 services. The Belgian health system is mainly financed by progressive direct taxation, proportional social security contributions related to income and alternative financing related to the consumption of goods and services [1].

In 2015, the Belgian Health System Performance Assessment report highlighted the satisfaction of the Belgian population with regards to their health system^2^ and according to the Euro Health Consumer Index 2016, which judges the satisfaction of the population regarding accessibility and quality of national health care, Belgium ranked fourth within EU15, i.e. after the Netherlands, Switzerland and Norway [2].

Since 1990, the Belgian federal and regional governments introduced multiple measures for tobacco control [3], cancer screening [4, 5], road injuries prevention [6, 7], healthier nutritional habits and physical activities [8] improved air quality [9] and care accessibility [10]. However, the Belgian Health System Performance Assessment report demonstrated that Belgium could do better in terms of health results. Several indicators of health promotion and lifestyle showed poor results and some of the mental health and mental health care indicators were alarming [2]. In addition, the Organisation for Economic Co-operation and Development (OECD) highlighted emerging issues related to inequalities in health and access to care, low investment in prevention, increase in some risk factors and waste in clinical care [11]. Health interview survey also highlighted that screening remained scattered and at low level in 2000 [12].

Despite these disappointing health results, health expenditure increased as a percentage of gross domestic product from 7.1% in 1990, to 10.4% in 2016, making Belgium’s health expenditure the fifth highest among all European countries in terms of percentage of gross domestic product [13]. Indeed, most of the costs in health seem to be linked to the way care is practiced in Belgium and the costs linked to investments in promotion and prevention actions and to ensure more equitable access are almost negligible [12].

In a context of pressure on social security as well as epidemiological and demographic changes, it seems crucial to focus effort on diseases and risk factors that cause the greatest burden on public health and to learn from the past and from other countries to invest better. Therefore, the objectives of this study were: 1) to perform an in-depth analysis of the Belgian health status changes between 1990 and 2016 using the Global Burden of Disease (GBD) 2016 study results for Belgium, and 2) to compare the Belgian health status with other European countries in 2016.

## Methods

### Overview

We collected results of the GBD 2016 study through the GBD results [14] and visualization tools [15]. Detailed information about data, approaches, statistical modelling, and metrics for the GBD 2016 study have been reported previously [16–21].

The GBD 2016 study used several metrics to quantify health impact of specific disease and injury causes – i.e., incidence, prevalence, mortality, Years of Life Lost due to premature mortality (YLLs), Years Lived with Disability (YLDs), Disability-Adjusted Life Years (DALYs) and Health-Adjusted Life Expectancy (HALE). The GBD 2016 study included also the Socio-Demographic Index (SDI), a summary measure of a geography’s socio-demographic development. SDI is based on average income per person, educational attainment, and total fertility rate. In this study we focused on mortality, YLLs, incidence, prevalence, YLDs and DALYs.

YLLs are expressing years of life lost and are computed by multiplying the number of deaths for a specific cause in each age-group by a reference life expectancy at that age. The life expectancy at birth in the GBD 2016 reference life table is 86 years for both sexes [22]. YLDs are calculated by multiplying the prevalence of sequelae by their disability weight (DW). DALYs are the sum of YLLs and YLDs [23, 24].

Deaths, YLLs, YLDs, and DALYs attributable to 84 risk factors or clusters of risk factors were also assessed in the GBD 2016 study [17]. In this study we investigated estimates of DALYs for all risks.

Although a complete set of age-specific, sex-specific, cause-specific, and geography-specific burden is available for the years 1990, 1995, 2000, 2005, 2010 and 2016 in the GBD estimations, we focused on the difference between 1990 and 2016, together with more detailed results for 2016.

### Benchmarking

We benchmarked Belgian GBD results with the other initial members of the European Union (EU15) – i.e., Austria, Denmark, Finland, France, Germany, Greece, Ireland, Italy, Luxembourg, Netherlands, Portugal, Spain, Sweden and the United Kingdom. Benchmarking with the other EU15 countries is most relevant to Belgian policy-makers; however, Belgium could easily be compared with (European) countries not included in our analysis using the GBD tools mentioned above. For diseases, injuries or risk factors, we ranked countries in 1990 and 2016 according to their age-standardized YLD, YLL, and DALY rates. Age-standardized rates in GBD are estimated using the GBD world population age standard. Briefly, they used the age-specific proportional distributions of all national locations from the World Population Prospects 2012 revision for all years from 2010 to 2035 and generated a standard population structure by taking the non-weighted mean across all the country-years [18]. Age-standardized rates allow comparing health outcomes across countries and are consequently often used for benchmarking studies [25, 26]. All rates presented in this manuscript therefore represent age-standardized rates, unless specified otherwise.

We evaluated if Belgian results were significantly different between 1990 and 2016 by visually determining whether the 95% uncertainty intervals (UIs) overlapped. We also estimated if Belgian performances were significantly different from the EU15 estimates from GBD 2016 by comparing the 95% UIs and ranked EU15 countries according to YLLs, YLDs and DALYs in 2016.

## Results

### Mortality and years of life lost

**Fig. 1:**
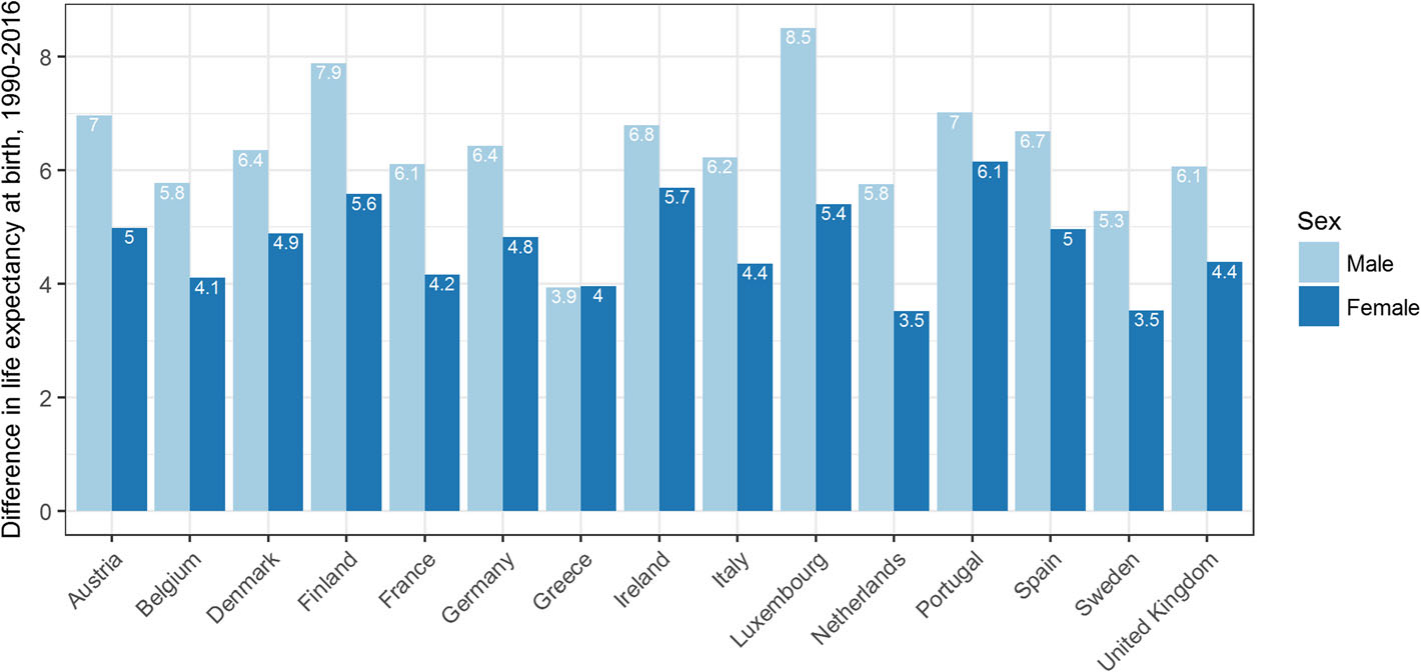
Difference in life expectancy at birth by sex, 1990–2016, EU15

Life expectancy (LE) at birth in the Belgium increased by 4.1 years (95% UI 3.5–4.9) for females and by 5.8 years (95% UI 5.1–6.4) for males from 1990 to 2016 (Fig. 1). Belgium did not perform differently than the EU15 average increase in LE at birth for females (+ 4.7 [95%UI 4.2–5.2]) and for males (+ 6.4 [95%UI 5.9–7.0]), nor for the EU15 average life expectancy at birth for females (Belgium: 83.4 years [95%UI 82.3–84.5] - EU15: 83.9 years [95%UI 83.1–84.8]) and males (Belgium: 78.4 years [95%UI 77.2–79.5] - EU15: 79.1 years [95%UI 78.2–80.1]) in 2016.

For women, Belgium ranked tenth in terms of mortality rate (MR) and did not perform significantly different than the EU15 female average in terms of MR in 2016 (Belgium: 408 [95% UI: 359–455] per 100,000; EU15: 389 [95% UI: 352–426] per 100,000). For men, Belgium ranked fourteenth and did not perform significantly different than the EU15 male average in terms of mortality rate (MR) in 2016 (Belgium: 642 [95% UI: 580–714] per 100,000; EU15: 605 [95% UI: 555–659] per 100,000).

In males, the major causes of mortality in Belgium in 2016 were ischemic heart disease (161 deaths [95% UI 143–180] per 100,000), lung, tracheal and bronchus cancer (95 deaths [95% UI 83–109] per 100,000) and Alzheimer’s disease (69 deaths [95% UI 56–84] per 100,000). Alzheimer’s disease (152 deaths [95% UI 123–183] per 100,000), ischemic heart disease (148 deaths [95% UI 131–168] per 100,000) and chronic obstructive pulmonary disease (COPD) (47 deaths [95% UI 41–54] per 100,000) were the leading causes of mortality in females (Additional file 1).

Deaths leading to YLLs, caused 15,115 YLLs [95% UI 14,649–15,566] per 100,000 in 1990 and 9221 YLLs [95% UI 8422–10,020] per 100,000 in Belgium in 2016. There was therefore a significant decrease of YLLs from 1990 to 2016 in Belgium.

The top three causes of YLL rates changed between 1990 and 2016 in females i.e., lung, tracheal and bronchus cancer was the third cause of YLL rates in 2016 but only the eighth in 1990. The major causes of YLL rates in females in 2016 in Belgium were ischemic heart disease (597 YLLs [95% UI 517–684] per 100,000), breast cancer (561 YLLs [95% 472–649] per 100,000), and tracheal, bronchus and lung cancer (440 YLLs [95% 372–511] per 100,000).

In males, although their relative contribution to total YLLs decreased drastically, the top three causes of YLL rates did not change between 1990 and 2016 – i.e., ischemic heart disease (1417 YLLs [95% UI 1246–1603] per 100,000), tracheal, bronchus and lung cancer (1178 YLLs [95% UI 1020–1350] per 100,000) and self-harm by other specified means (870 YLLs [95% UI 711–1173] per 100,000) were still in the top of the ranking.

**Fig. 2:**
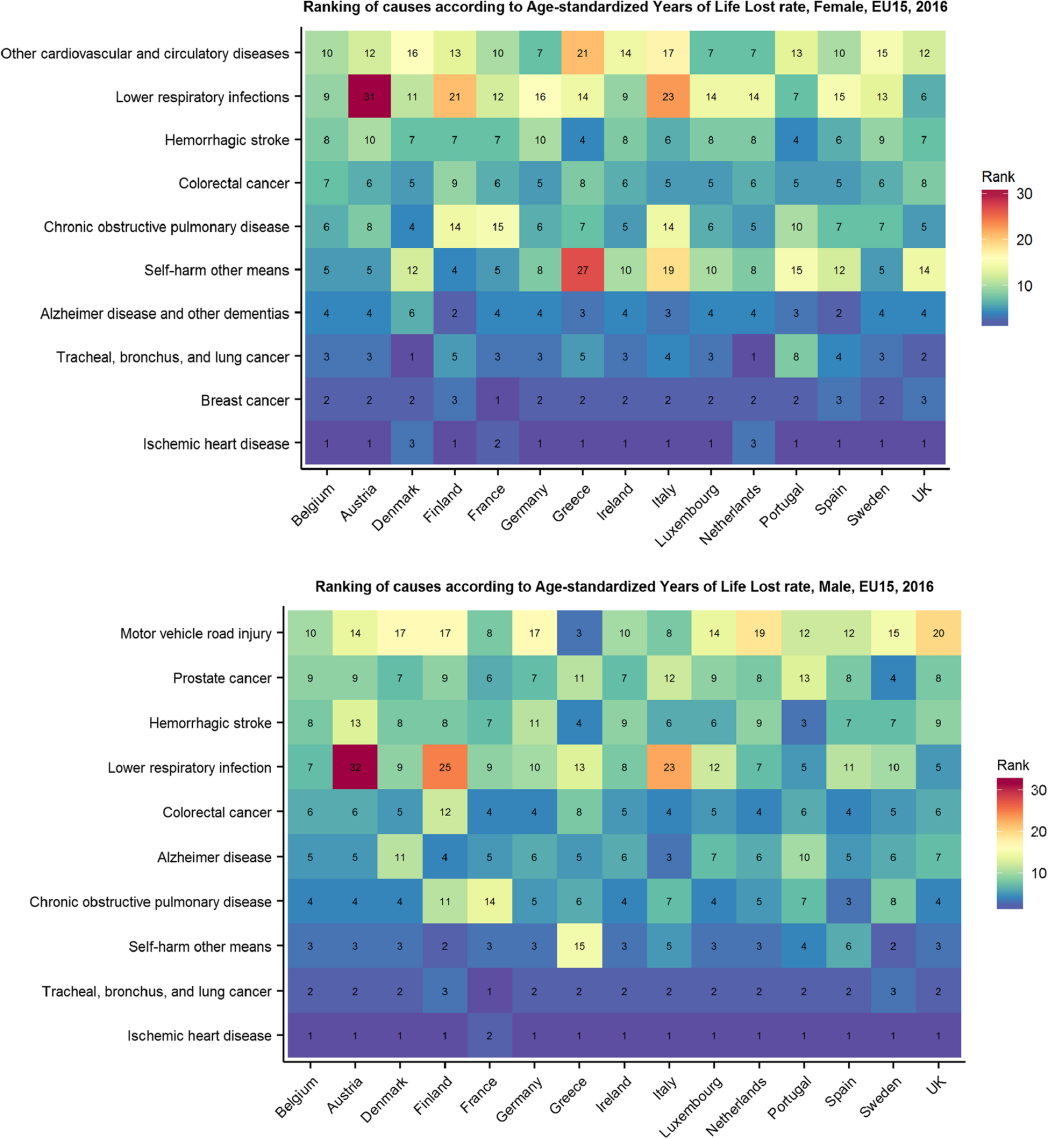
Ranking (decreasing) of causes according to age-standardized Years of Life Lost rate, by sex, EU15, 2016. Other cardiovascular diseases = cardiovascular and circulatory diseases different than rheumatic heart disease, ischemic heart disease, cerebrovascular disease, hypertensive heart disease cardiomyopathy and myocarditis, atrial fibrillation and flutter, aortic aneurysm, peripheral vascular disease and endocarditis

The top three causes of YLLs in females and males were consistent across all EU15 countries. (Fig. 2).

**Fig. 3:**
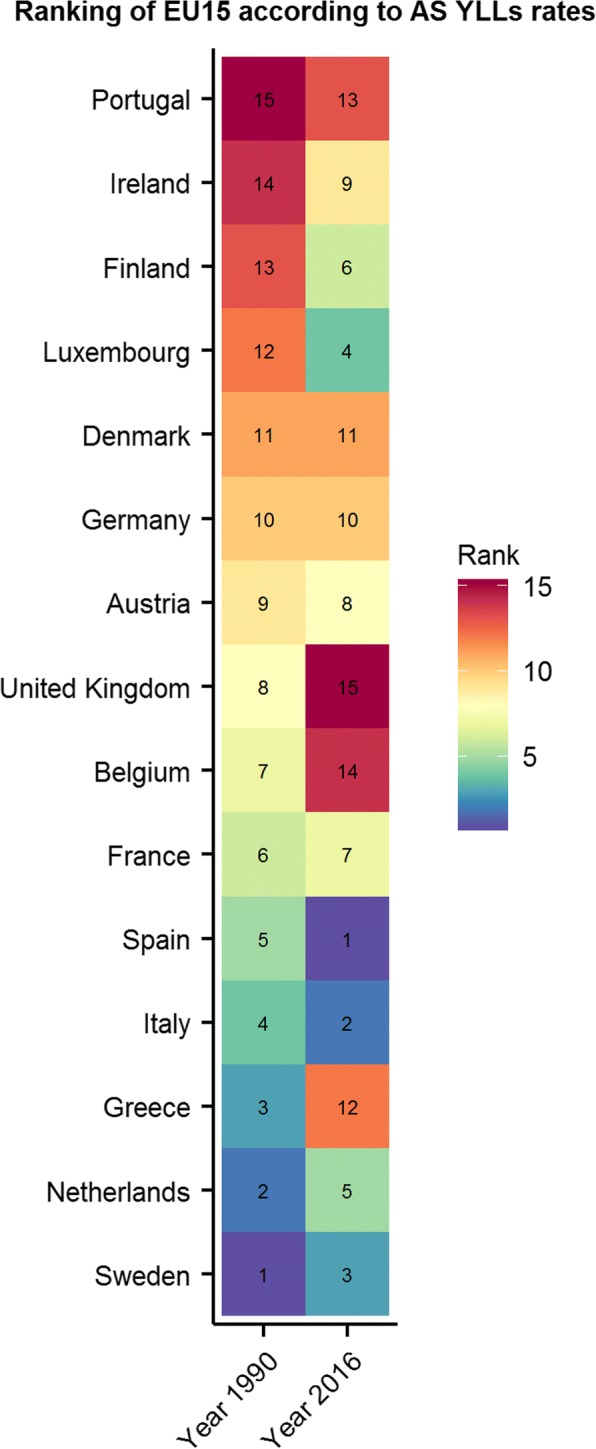
Ranking (ascending) of EU15 countries according to age-standardised Years of life Lost (YLLs) per 100,000, 1990–2016

Although Belgium did not perform significantly worse than the EU15 countries in terms of age-standardized YLL rates in 2016 (Belgium: 9221 YLLs [95%UI 8422–10,020] per 100,000 – EU15: 8531 YLLs [95%UI 7939–9166] per 100,000), Belgium fell drastically in the EU15 ranking between 1990 and 2016. Indeed, Belgium ranked 14th in terms of YLLs per 100,000 in 2016, i.e., 7 places less than in 1990. Spain had the lowest YLLs (7314 YLLs [95%UI 6967–7681] per 100,000) while UK was in last position (9222 YLLs [95%UI 9084–9372] per 100,000) in 2016 (Fig. 3).

**Fig. 4:**
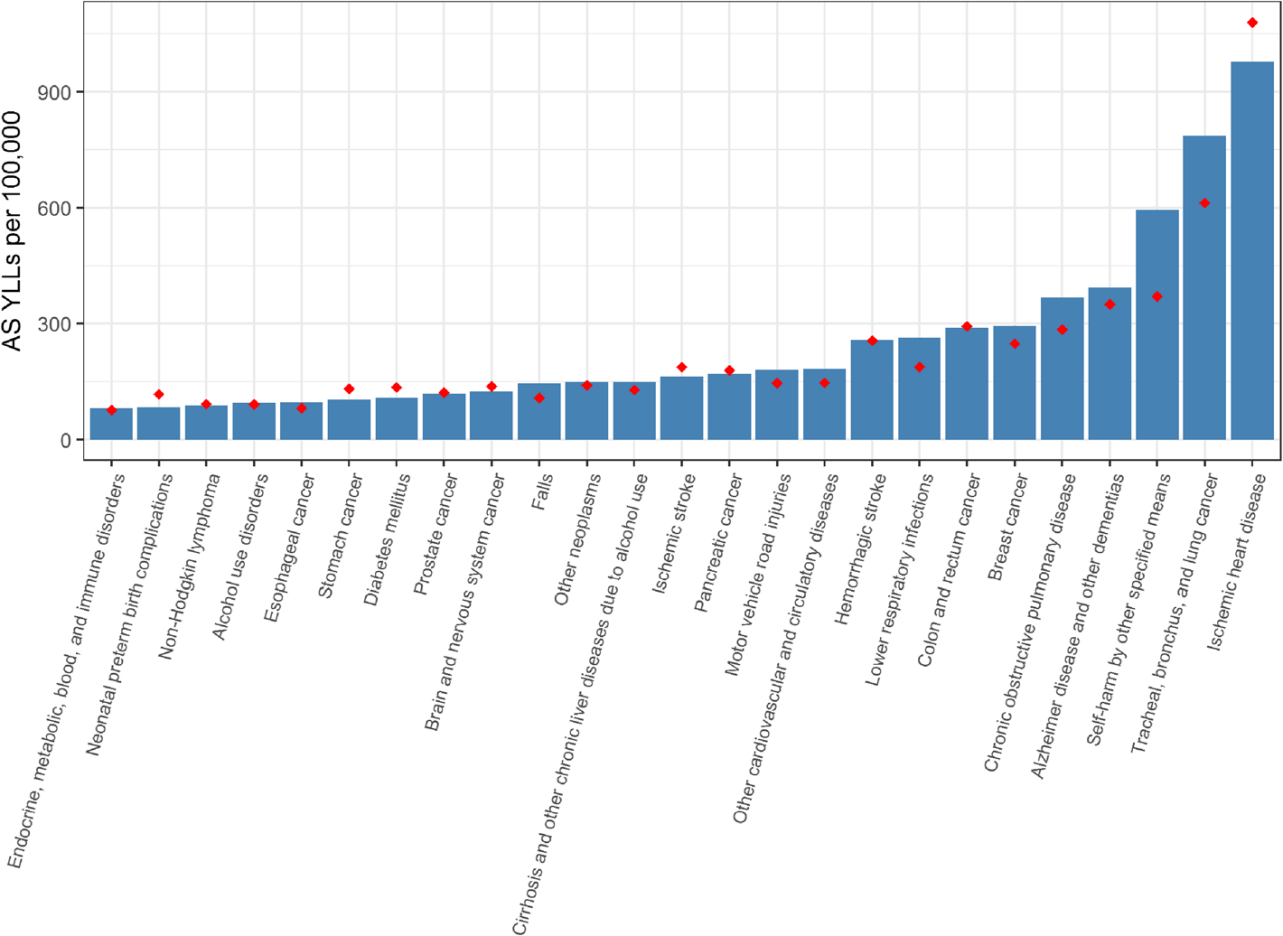
Top 25 causes of age-standardised (AS) Years of life lost (YLLs) per 100,000, 2016, Belgium. Red dots represent EU15 average AS YLLs per 100,000 in 2016. Other neoplasms = Neoplasms different than lip and oral cavity cancer, nasopharynx cancer, oesophageal cancer, stomach cancer, colon and rectum cancer, liver cancer, gallbladder and biliary tract cancer, pancreatic cancer, larynx cancer, tracheal, bronchus, and lung cancer, malignant skin melanoma, non-melanoma skin cancer, breast cancer, cervical cancer, uterine cancer, ovarian cancer, prostate cancer, testicular cancer, kidney cancer, bladder cancer, brain and nervous system cancer, thyroid cancer, mesothelioma, Hodgkin lymphoma, multiple myeloma and leukaemia

Compared to the EU15, Belgium had significantly lower YLLs for stomach cancer (Belgium: 105 YLLs [95%UI 97–112] per 100,000; EU15: 132 YLLs [95%UI 120–145] per 100,000) and diabetes mellitus (Belgium: 108 YLLs [95%UI 96–120] per 100,000; EU15: 131 YLLs [95%UI 122–150] per 100,000). Conversely, Belgium had significantly higher YLLs for lower respiratory infections (Belgium: 264 YLLs [95%UI 231–301] per 100,000; EU15: 188 YLLs [95%UI 168–212] per 100,000), for chronic obstructive pulmonary disease (Belgium: 368 YLLs [95%UI 331–407] per 100,000; EU15: 285 YLLs [95%UI 258–316] per 100,000) and tracheal, bronchus, and lung cancer (Belgium: 785 YLLs [95%UI 699–879] per 100,000; EU15: 613 YLLs [95%UI 556–674] per 100,000) (Fig. 4).

### Disability

Disabilities resulted in 10,833 [95% UI 8060–14,038] YLDs per 100,000 in 1990 and 10,786 [95% UI 8005–13,981] YLDs per 100,000 in 2016 (Additional file 1). There was no significant difference of YLDs rates between 1990 and 2016 in Belgium.

The major causes of YLDs in 2016 in males were low back pain (1127 YLDs [95% UI 789–1516] per 100,000), migraine (523 YLDs [95% UI 334–736] per 100,000) and falls (450 YLDs [95% UI 304–631] per 100,000) (Additional file 1). For all age-groups in males, the contributions of major depression (+ 24%), benign prostatic hyperplasia (+ 18%), and falls (+ 12%), to total YLDs rates rose from 1990 to 2016.

In females low back pain (1403 YLDs [95% UI 984–1863] per 100,000), migraine (1042 YLDs [95% UI 662–1464] per 100,000) and major depression (702 YLDs [95% UI 479–962] per 100,000) caused most of the YLDs in 2016 (Additional file 1). For all age-groups in females, the contributions of major depression (+ 22%) and falls (+ 18%) rose to total YLDs rates from 1990 to 2016.

In 2016, females contributed slightly more YLDs (11,515 YLD [95% UI 8584–14,952] per 100,000) than males (10,070 YLD [95% UI 7441–13,034] per 100,000).

For both men and women in 2016, the group over 80 years showed the highest YLD rate (105,949 YLD [95% UI 80,256–134,106] per 100,000), followed by the 75–79 years (24,813 YLD [95% UI 18,777–31,605] per 100,000) and 70–74 years (21,775 YLD [95% UI 16,320–28,189] per 100,000) groups. The groups of < 1 year (3424 YLD [95% UI 2384–4751] per 100,000) had the lowest burden in terms of YLDs in both sexes in 2016.

Compared to 1990, Belgium lost a place in 2016 and is the median of the EU countries, i.e. 8th in terms of YLDs rates. However, no significant difference was observed of the overall YLDs rates in 1990 and 2016 between Belgium and EU15 (Additional file 1) and 2016 cause specific YLD’s (Fig. 4).

### Disability-adjusted life years

All causes of diseases resulted in 25,948 age-standardized Disability-Adjusted Life Years (DALYs) [95% UI 23,160–29,223] per 100,000 in 1990 and 20,027 DALYs [95% UI 17,333–22,747] per 100,000 in 2016. There was no significant difference of DALY rates between 1990 and 2016 in Belgium. In 1990, 42% of the total DALYs was caused by YLDs and in 2016, 54% of the total DALYs was caused by YLDs.

Despite an impressive decrease of DALYs between 1990 and 2016 (− 2380 DALYs per 100,000), ischemic heart disease remained the number one cause of DALYs in males in 2016 (8.5% [95% 7.6–9.5 UI] of total DALYs). Lung, tracheal and bronchus cancer (6.4% [95% 5.5–7.3 UI] of total DALYs) and low back pain (4.8% [95% 3.7–5.9 UI] of total DALYs) were second and third major causes of DALYs in males in 2016 (Additional file 1). DALYs per 100,000 caused by major depressive disorder (+ 77 DALYs per 100,000), alcohol use disorders (+ 44 DALYs per 100,000) and falls (+ 23 DALYs per 100,000) increased between 1990 and 2016 in males in Belgium (Table 1).

**Table 1:** Age-standardized Disability-Adjusted Life Years per 100,000 by cause, males, 1990 and 2016, Belgium

	Belgium, 1990				Belgium, 2016				
Causes	DALY rank	DALY ^a^ rate	LB 95% UI	UB 95% UI	DALY rank	DALY^a^ rate	LB 95% UI	UB 95% UI	Absolute DALYs changes^a^
Ischemic heart disease	1	3936	3716	4159	1	1556	1384	1748	−2380
Tracheal, bronchus, and lung cancer	2	2140	1987	2296	2	1195	1038	1367	− 945
Low back pain	3	1143	795	1512	3	1127	789	1516	−17
Self-harm by other specified means	5	954	826	1381	4	879	720	1186	−75
Chronic obstructive pulmonary disease	4	1115	1032	1204	5	666	578	755	−449
Falls	9	617	483	789	6	640	481	827	23
Migraine	11	527	337	753	7	523	334	736	−4
Alzheimer disease and other dementias	13	491	410	587	8	460	382	558	−31
Age-related and other hearing loss	18	434	296	632	9	425	288	625	−9
Major depressive disorder	23	334	225	456	10	411	281	566	77
Neck pain	20	406	270	578	11	407	273	583	0
Diabetes mellitus	16	446	361	547	12	392	304	496	−53
Colon and rectum cancer	10	535	495	580	13	381	326	442	−154
Other cardiovascular and circulatory diseases	17	442	362	539	14	374	314	457	−67
Lower respiratory infections	19	429	387	470	15	362	304	428	−67
Anxiety disorders	21	352	244	476	16	353	245	479	0
Hemorrhagic stroke	8	648	598	701	17	338	292	388	− 311
Motor vehicle road injuries	6	902	755	998	18	330	274	414	− 573
Alcohol use disorders	25	282	224	359	19	327	263	401	44
Ischemic stroke	7	723	660	792	20	325	277	374	− 398

^a^age-standardized DALY per 100,000

In females, DALYs caused by major depressive disorder increased drastically between 1990 and 2016 (+ 127 DALYs per 100,000) and appeared as the third cause of DALYs in 2016. The major causes of DALYs in females in 2016 were low back pain (6.7% [95% 5.4–8.0 UI] of total DALYs) and migraine (5.7% [95% 4.9–6.6 UI] of total DALYs) (Table 2). DALYs caused by falls (+ 53 DALYs per 100,000) also increased between 1990 and 2016 in females in Belgium.

**Table 2:** Age-standardized Disability-Adjusted Life Years per 100,000 by cause, females, 1990 and 2016, Belgium

	Belgium, 1990				Belgium, 2016				
Causes	DALY rank	DALY ^a^ rate	LB 95% UI	UB 95% UI	DALY rank	DALY^a^ rate	LB 95% UI	UB 95% UI	Absolute DALYs changes^a^
Low back pain	2	1423	997	1888	1	1403	984	1863	−19
Migraine	3	1048	667	1486	2	1042	662	1464	−6
Major depressive disorder	7	575	392	787	3	702	479	962	127
Ischemic heart disease	1	1781	1684	1883	4	680	598	770	− 1101
Breast cancer	4	920	862	989	5	610	521	699	− 310
Anxiety disorders	5	584	401	799	6	582	402	799	−2
Neck pain	8	563	379	799	7	563	380	803	0
Alzheimer disease and other dementias	6	577	493	676	8	526	434	636	−51
Falls	11	460	349	600	9	513	386	670	53
Tracheal, bronchus, and lung cancer	23	299	274	325	10	447	378	518	148
Chronic obstructive pulmonary disease	16	365	336	395	11	353	309	399	−12
Age-related and other hearing loss	17	352	238	512	12	348	237	508	−4
Self-harm by other specified means	13	419	388	450	13	335	282	392	−84
Acne vulgaris	21	314	208	453	14	332	221	482	18
Other cardiovascular and circulatory diseases	14	401	325	488	15	323	265	390	−78
Other musculoskeletal disorders	22	299	200	424	16	319	218	453	20
Diabetes mellitus	12	431	356	521	17	300	229	385	−131
Bipolar disorder	24	255	130	442	18	255	127	450	0
Hemorrhagic stroke	10	512	474	554	19	255	219	292	−257
Ischemic stroke	9	541	490	595	20	248	208	291	− 293

^a^age-standardized DALY per 100,000

Low back pain and ischemic heart disease were the diseases that caused most of the DALYs in females and males respectively in all European countries in 2016.

**Fig. 5:**
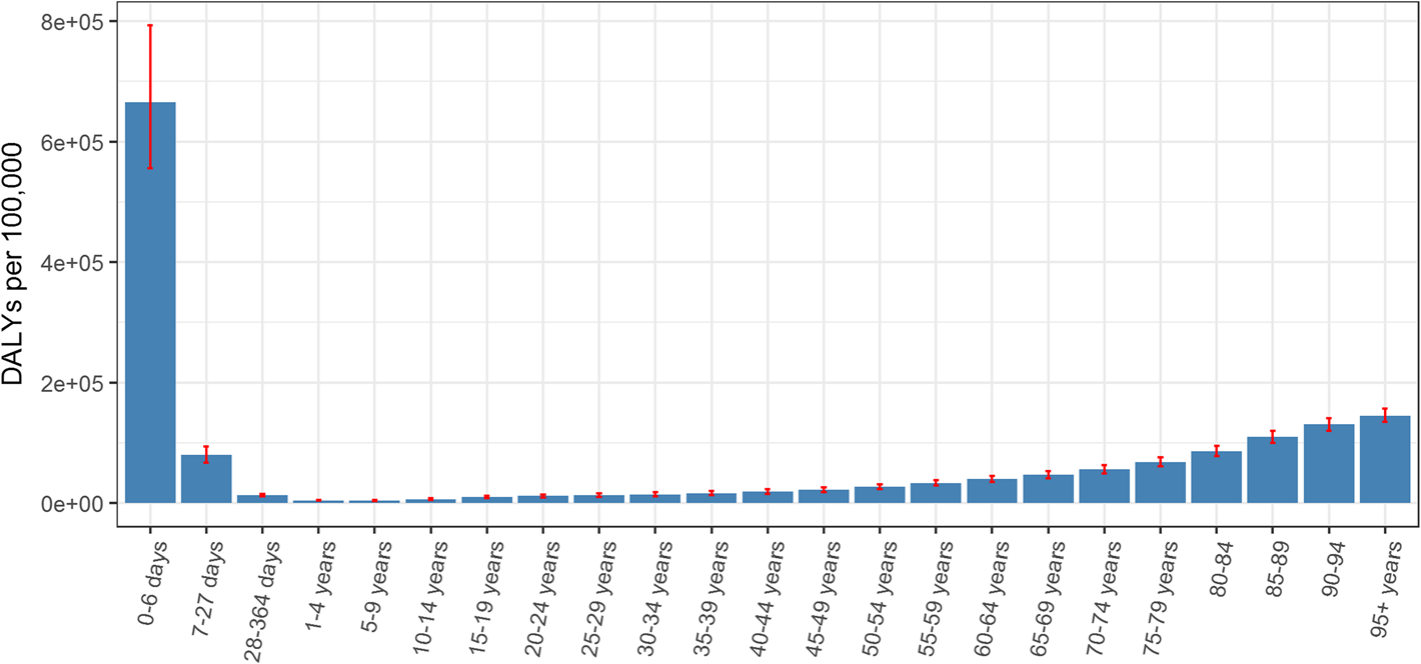
Disability-Adjusted Life Years per 100,000 and 95% UI by age-group, 2016, Belgium

For both men and women in 2016, the youngest age group of infants aged 0–6 days showed the highest DALY rate (665,481 DALYs [95% UI 555,762–793,147] per 100,000), followed by the 80+ years (471,865 DALYs [95% UI 432,866 –512,650] per 100,000) and 7–27 days (79,437 DALYs [95% UI 67,225–93,449] per 100,000) groups (Fig. 5).

**Fig. 6:**
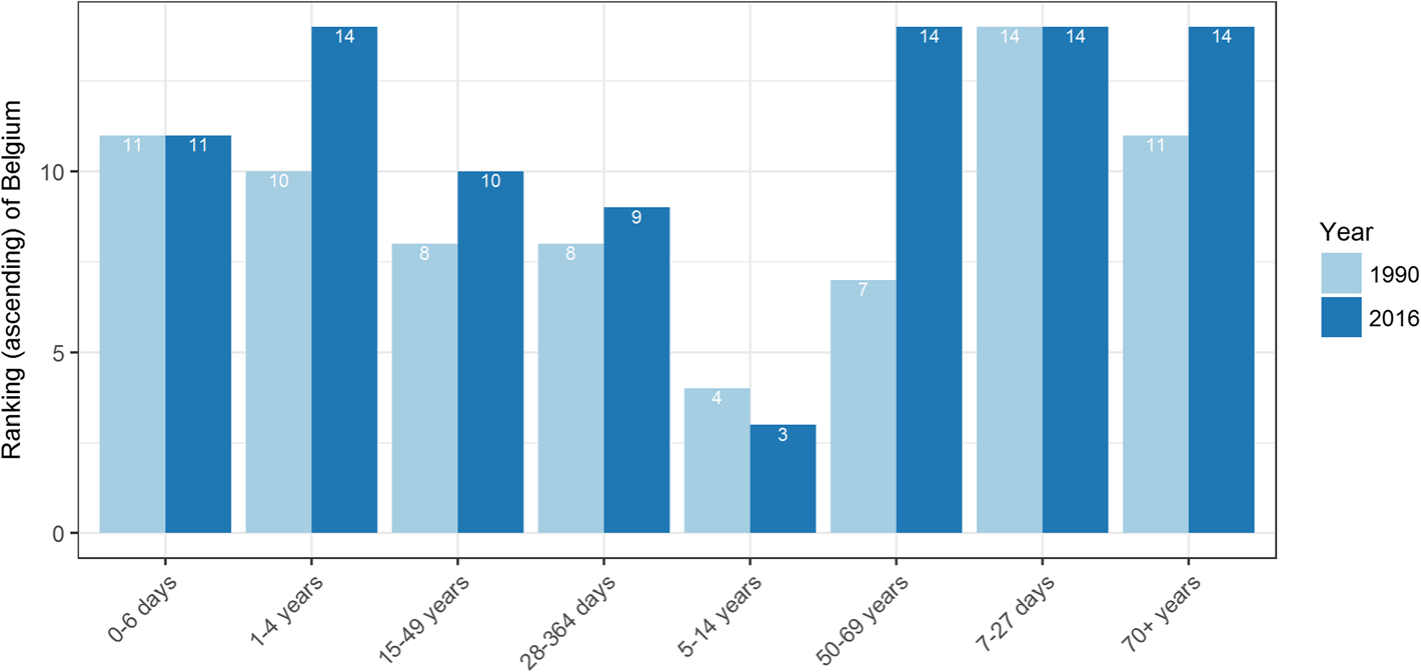
Ranking (ascending) of Belgium, age-standardized Disability-Adjusted Life Years per 100,000 among EU15, both sexes, 1990 and 2016

Although we did not observe significant differences of DALYs rates between Belgium and the EU15 in 1990 (Belgium: 25,498 DALYs [95% UI 23,160–29,223] per 100,000; EU15: 26,027 DALYs [95% UI: 23,154–29,269] per 100,000) and in 2016 (Belgium: 20,006 DALYs [95% UI 17,108–23,233] per 100,000; EU15: 19,249 DALYs [95% UI: 16,374–22,432] per 100,000), the ranking of Belgium among the EU15 in terms of DALYs in both sexes was worse in 2016 than in 1990. Indeed Belgium moved from 7th to 14th place in terms of DALYs per 100,000 and for all age groups, except for 0–6 days, 7–27 days and 5–14 years groups, ranking became worse (Fig. 6).

Finally, Belgium ranked twelfth in terms of difference of DALYs per 100,000 from 1990 to 2016, which means that despite improving of health between 1990 and 2016, Belgium did worse than most of the EU15 countries. In Belgium most of the difference of DALYs rate from 1990 to 2016 was linked to a reduction in YLLs rate (99%) (Table 3).

**Table 3:** Age-standardized Disability-Adjusted Life Years per 100,000, difference between 1990 and 2016, EU15

Country	Difference DALYs 90–16 per 100.000	LB 95%UI	UB 95% UI	% YLLs in Difference DALYs 90–16	% YLDs in Difference DALYs 90–16
Portugal	− 9131	−9221	− 9232	96%	4%
Luxembourg	− 8813	− 8749	− 8956	94%	6%
Finland	− 8345	− 8212	− 8494	95%	5%
Ireland	− 7610	− 7569	− 7548	98%	2%
Austria	− 7210	− 7188	− 7145	97%	3%
Germany	− 6962	− 7020	− 6780	96%	4%
Spain	− 6942	− 6880	− 7212	95%	5%
Denmark	− 6939	− 6903	− 6940	101%	−1%
Italy	− 6433	− 6400	− 6580	94%	6%
United Kingdom	− 6248	− 6198	− 6328	96%	4%
France	− 5957	− 5974	− 6005	95%	5%
Belgium	− 5942	− 6052	− 5990	99%	1%
Netherlands	− 5434	− 5631	− 5419	97%	3%
Sweden	− 4962	− 4992	− 4939	99%	1%
Greece	− 4742	− 4720	− 4987	96%	4%

### Risk factors

In 1990, the considered risk factors accounted for 7195 DALYs [95% UI: 6640–7818] per 100,000 in females and 14,853 DALYs [95% UI: 13,931–15,872] per 100,000 in males. In 2016, the number of DALYs linked to risk factors decreased in both females (5114 DALYs [95% UI: 4467–5796] per 100,000) and males (8975 DALYs [95% UI: 7948–10,179] per 100,000). Proportions of DALYs attributed to risk factors also decreased between 1990 and 2016 in females (− 4% of total DALYs) and males (− 8% of total DALYs).

**Fig. 7:**
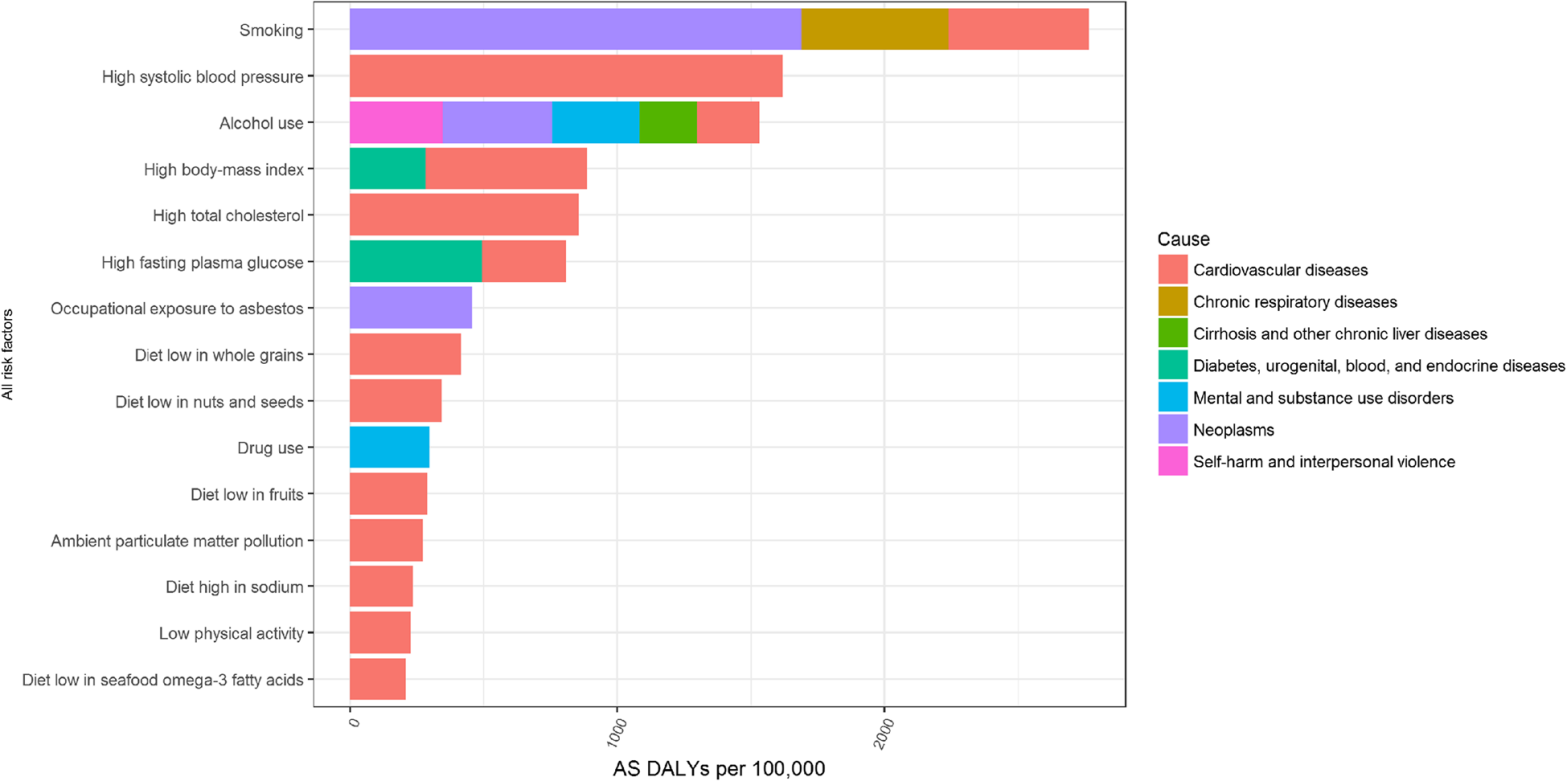
Risk factors of age-standardized Disability-Adjusted Life Years per 100,000, males, 2016, Belgium

In males, the leading risk factors of age-standardized DALYs in Belgium in 2016 were smoking (14% of total DALYs), alcohol use (9% of total DALYs) and high systolic blood pressure (8% of total DALYs) and risk factors resulted mainly in cardiovascular diseases and neoplasms (Fig. 7).

**Fig. 8:**
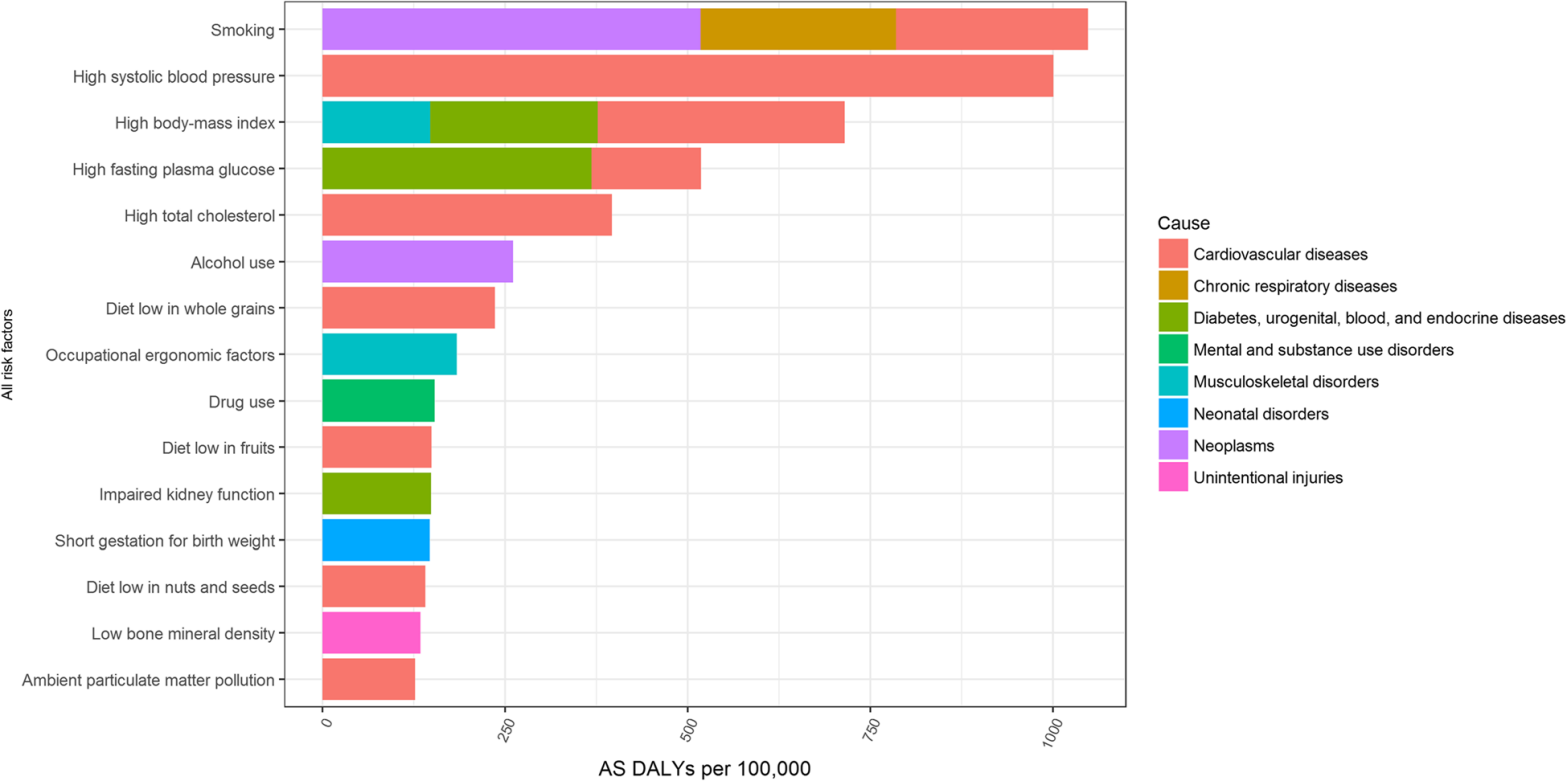
Risk factors of age-standardized Disability-Adjusted Life Years per 100,000, females, 2016, Belgium

In females, smoking (7% of total DALYs), high systolic blood pressure (6% of total DALYs) and high body mass index (6% of total DALYs) were the major risk factors of DALYs in 2016 and caused mainly cardiovascular diseases, neoplasms and diabetes (Fig. 8). Additional results on risk factors in Belgium are available in the Additional file 1**.**

## Discussion

Overall the health status of the Belgian population improved between 1990 and 2016 but to a lesser extent compared to the other EU15 countries. Belgium ranked worse in 2016 than in 1990 among the EU15 countries. This study further highlighted some important changes in health in Belgium between 1990 and 2016.

First, LE at birth increased in Belgium between 1990 and 2016 (4.1 years (95% UI 3.5–4.9) for females and 5.8 years (95% UI 5.1–6.4) for males) and is not significantly below the average EU15. We observed the same pattern for death as for LE at birth, i.e. a significant improvement between 1990 and 2016 in Belgium and an average MR not different than the EU average in 2016. Alzheimer’s disease and ischemic heart disease are a major cause of death in both sexes in Belgium.

Second, there was a significant decrease of YLLs from 1990 to 2016 in Belgium. Ischemic heart disease (IHD), breast cancer and lung, tracheal and bronchus cancer were the leading causes of premature mortality in females in Belgium in 2016 and IHD, lung, tracheal and bronchus cancer and self-harm were the main causes of the premature deaths in males. The Belgian Health Care Knowledge Center also published alarming conclusions for mental health care and revealed that the Belgian suicide rate (18.3 per 100,000 population) was considerably higher than in other European countries (10.6 for EU15 countries) [2]. Bossuyt and colleagues also reported that between 1993 and 1995 and 2000–2001, the incidence of suicide increased among men in Belgium [27] and Vancayseele and colleagues reported the highest rates of suicide in Flanders among adolescents and young adults and in 2008, i.e. related to the beginning of the economic crisis [28].

Third, even if Belgium did not seem to perform significantly worse than the EU15 in terms of YLLs in 2016, we observed that Belgium fell drastically in the EU15 ranking from 1990 (7th rank) to 2016 (14th rank) and that Belgium had significantly higher YLLs rates than EU15 for some specific causes as lower respiratory infections, chronic obstructive pulmonary disease and tracheal, bronchus, and lung cancer. This result has also been supported by the Belgian Health Care Knowledge Center that highlighted that premature death was relatively high in Belgium in 2015 and that it was particularly the case for causes of death preventable through public policies (for instance deaths from lung cancer) [2].

Fourth, YLDs were mainly driven by low back pain, migraine, major depressive disorders and falls in 2016 in Belgium. There was no major change in YLDs rates in Belgium between 1990 and 2016 for both males and females and we did not observe significant difference of YLDs rates between Belgium and EU15.

Fifth, we did not observe major differences in DALY rates between 1990 and 2016 and the proportion of the burden attributed to YLDs increased from 1990 to 2016 in Belgium. In other words, most of the burden of diseases in Belgium in 2016 is caused by years lived with disabilities. This could be explained by increasing of life expectancy and of number of people living with comorbidities. We also highlighted an important increasing of DALYs linked to major depressive disorders in both sexes in Belgium from 1990 to 2016 and that the ranking of Belgium among the EU15 in terms of DALYs in both sexes was drastically worse in 2016 (14th rank) than in 1990 (7th rank).

Finally, although we observed that DALYs caused by risk factors decreased significantly in Belgium from 1990 to 2016, we also highlighted that smoking, high blood pressure, high body mass index and alcohol use were still the major risk factors of DALYs in Belgium in 2016. OECD also highlighted that alcohol consumption was a major risk factor of death and classified Belgium 2nd highest rate of alcohol consumption across the EU28 [11]. Van Oyen and colleagues concluded that smoking kills and shortens both life without and life with disability in Belgium [29] and Yokota and colleagues showed an increasing trend of the disability prevalence and different contributors to the disability burden across smoking categories in Belgium [30].

Although this is the first in-depth analysis of the Belgian GBD results, these results have to be interpreted carefully and cannot replace a national burden of disease study because they suffer from all limitations of GBD 2016 estimates already discussed widely and in detail elsewhere [16–19]. We summarize the relevant limitations for Belgium and EU15 countries focusing on data sources and model used.

First, in the GBD 2016 study there were no data for some sequelae and for some or even many countries making estimates for a country and between countries over time challenging. For Belgium, 520 data sources were reported in Global Health Data exchange (GHDx) and only one referred to the year 2016. In other words, GBD 2016 estimates for Belgium were largely based on data from previous years or from other countries, even non-European countries. Among the EU15 countries, UK (*n* = 2998), Italy (*n* = 1754), Sweden (*n* = 1649), France (*n*=1182) and the Netherlands (*n* = 1090) had most of the data sources reported in GHDx. Although Belgium is probably behind in terms of health data collection compared to other EU15 countries, some available data sources as Belgian Cancer Registry or Belgian Diabetes Registry were not used by the Institute for Health Metrics and Evaluation (IHME) in the GBD 2016 estimates. Presenting results for the time window 1990 to 2016 could mask very recent changes, e.g. in the last ten years; however, we decided to present results for this time window because, as demonstrated above, the relevance of GBD’s estimates for the most recent years is not always guaranteed. Changes in data quality over time can also occur and can have an impact on the GBD estimates, even if all estimates are generated within a single model. As GBD 2016 Belgian results are based on data from other countries and complex modeling, it is important to not solely relying on GBD estimates, to increase investments in national health monitoring and to generate national health status and burden of disease estimates [25].

Second, as detailed elsewhere [26, 31], Bayesian models were used to estimate health metrics of conditions in each country, age, sex and year. The nature of this estimation process means that, without data or powerful covariates, estimated variance might be smaller than the real variance. Results for Belgium have been informed by many available data sources such as vital registration data, surveillance report or studies on specific diseases. UIs provide some information about the extent of available information for Belgium.

It is also noteworthy that there are international agencies that publish EU15 health status reports such as OECD, European Commission, World Health Organization (WHO), and the WHO European Regional Office. The main advantage of the IHME initiative is that it generates internally consistent estimates, thus allowing for comparisons across countries. However, external validity is not always guaranteed, as evidenced by the differences between different reports.

Finally, comparing overlapping UIs is not a very robust method to estimate differences of health status across countries and year. Indeed, when UIs do not overlap, it is safe to conclude that the differences are significantly different; however, when UIs do overlap, the difference can still be statistically significant. In other words, it means that there may still be differences of health status between EU15 countries even if UIs overlap. However as GBD results are based on simulation, comparing UIs is the only possible method.

Between 1990 and 2016, several health policies have been initiated in Belgium, for instance, the introduction in 1999 of a Global Medical Dossier for patients to strengthen the role of primary care, the extension of preferential reimbursements to all persons under a fixed income limit, the introduction in 2007 of reimbursements for analgesic drugs and bandages, the launch in 2008 of a cancer plan and a national action plan for alcohol, the introduction in 2009 of the pathway for diabetes follow-up and chronic renal failure or prohibition in 2011 of smoking in closed public places. Although these measures may have contributed to an improvement of the health of the Belgian population, we also observed that Belgium performed less well than other EU15 countries.

Multiple other factors than demographic changes and policies may have contributed to the fact that the average Belgian health status became worse than other EU15 countries, for instance health competencies distributed among different levels of power or the education system. However, Belgian health policies and initiatives should make additional efforts to be better oriented, i.e. better focused on risks factors and on diseases that caused most of the health burden. In addition, more efforts have to be made to define health goals or to focus prevention actions in at risk groups.

Inequality in health across EU15 should also be further studied, using for example the SDI index available in the 2016 GBD study.

In 2015, the Belgian government approved a reform of health care funding and especially of the hospital funding system. In such a context, future studies need to monitor the situation and assess the impact of these changes.

## Conclusion

Even though YLLs due to premature deaths decreased between 1990 and 2016 in Belgium, Belgium’s ranking among the EU15 in terms of YLLs and DALYs decreased from 1990 to 2016. Significant health gains appear possible by acting on risk factors directly linked to a significant part of the Belgian burden of diseases, i.e., alcohol and tobacco consumption, and high body mass index. Care management of people with chronic disease or long-term severe disease sequelae must also receive special attention, because they are carrying a heavy burden in Belgium. National burden of disease estimates can help defining Belgian health targets and are necessary as external validity of GBD results is not always guaranteed.

## Additional file

Additional file 1: Includes six additional figures and additional results on risk factors linked with DALYs in Belgium in 2016. Figure S9 in the Additional file 1 represents the top 15 causes of age-standardized (AS) deaths per 100,000 by sex in 2016 in Belgium. Fig. S10 represents the AS YLDs per 100,000 in EU15 in 1990 and 2016. Fig. S11 represents the ranking (descending) and contribution of health states by AS YLDs per 100,000 in 1990 and 2016 in males in Belgium. Fig. S12 represents the ranking (descending) and contribution of health states by AS YLDs per 100,000 in 1990 and 2016 in females in Belgium. Finally, we presented additional results on risk factors linked with DALYs in Belgium in 2016 and Fig. S13 and S14 represent selected disorders attributable to risk factors linked with DALYs in males and females in 2016 in Belgium. (DOCX 474 kb)

## Abbreviations

AS: Age-standardized
COPD: Chronic obstructive pulmonary disease
DALY: Disability-adjusted life year
DW: Disability weight
EU: European Union
GBD: Global burden of disease
GHDx: Global health data exchange
HALE: Health-adjusted life expectancy
IHD: Ischemic heart disease
IHME: Institute for health metrics and evaluation
LE: Life expectancy
MR: Mortality rate
OECD: Organisation for economic co-operation and development
SDI: Socio-demographic index
UI: Uncertainty interval
UK: United Kingdom
WHO: World Health Organization
YLD: Years lived with disability
YLL: Year of life lost

## References

[1] Gerkens E, Merkur S. Belgium: Financial protection and equity in financing. 2010. Available on: http://www.hspm.org/searchandcompare.aspx.

[2] Vrijens F, Renard F, Camberlin C, Desomer A, Dubois CPJ, et al. Performance of the belgian health system- report 2015. 2016. Available on: https://kce.fgov.be/sites/default/files/atoms/files/KCE_259C_performancereport2015_0.pdf. 10.1016/j.healthpol.2013.06.010 23927845

[3] Joossens L RM. Progress in Tob Control in 30 European Countries from 2005 to 2007. 2007. Available on: http://lernbuch.lernetz.ch/ews/tabaction_fr/lightbox/excursion_chapter4/Kap04_Tabakpraevention_Laendervergleich_fr_en.pdf.

[4] Van Kerrebroeck H, Makar A (2016). Cervical cancer screening in Belgium and overscreening of adolescents. Eur J Cancer Prev.

[5] Ponti A, Anttila A, Ronco G, Senore C. Cancer Screening in the European Union. Report on the implementation of the Council Recommendation on cancer Screening. 2017. Available on: https://ec.europa.eu/health/sites/health/files/major_chronic_diseases/docs/2017_cancerscreening_2ndreportimplementation_en.pdf.

[6] Focant N. Drinking and driving. Do we do it too much? National behavioural survey “driving under the influence of alcohol” 2015. BIVV-IBSR, 2016.

[7] Lequeux Q (2016). What about the seatbelt use? Results of the seatbelt behaviour measurement 2015.

[8] Cuypers K LT, Bel S. Introduction et méthodologie. Dans : Lebacq T, Teppers E (éd.). Enquête de consommation alimentaire 2014–2015. Rapport 1. 2015.

[9] Fierens F, Vanpoucke C, Adriaenssens S, Trimpeneers E, Peeters O. Brasseur O, et al. Annual report Air Quality in Belgium. 2011:2011.

[10] Dewulf B, Neutens T, De Weerdt Y, Van de Weghe N (2013). Accessibility to primary health care in Belgium: an evaluation of policies awarding financial assistance in shortage areas. BMC Fam Pract.

[11] Pearson M. Performance of the Belgium health system in international comparison. Available on: https://www.health.belgium.be/sites/default/files/uploads/fields/fpshealth_theme_file/presentation_mark_pearson_belgium_health_system_performance_final.pdf. 2017.

[12] https://his.wiv-isp.be/SitePages/Home.aspx

[13] OECD. Health at a glance. OECD inidactors. 2017. Available on https://www.oecd.org/els/health-systems/Health-at-a-Glance-2017-Chartset.pdf.

[14] http://ghdx.healthdata.org/gbd-results-tool.

[15] https://vizhub.healthdata.org/gbd-compare/

[16] Collaborators GDH (2017). Global, regional, and national disability-adjusted life-years (DALYs) for 333 diseases and injuries and healthy life expectancy (HALE) for 195 countries and territories, 1990-2016: a systematic analysis for the global burden of disease study 2016. Lancet.

[17] Collaborators GBDRF (2017). Global, regional, and national comparative risk assessment of 84 behavioural, environmental and occupational, and metabolic risks or clusters of risks, 1990-2016: a systematic analysis for the global burden of disease study 2016. Lancet.

[18] Disease GBD, Injury I, Prevalence C (2017). Global, regional, and national incidence, prevalence, and years lived with disability for 328 diseases and injuries for 195 countries, 1990-2016: a systematic analysis for the global burden of disease study 2016. Lancet.

[19] Collaborators GBDCoD (2017). Global, regional, and national age-sex specific mortality for 264 causes of death, 1980-2016: a systematic analysis for the global burden of disease study 2016. Lancet.

[20] Collaborators GBDM (2017). Global, regional, and national under-5 mortality, adult mortality, age-specific mortality, and life expectancy, 1970-2016: a systematic analysis for the global burden of disease study 2016. Lancet.

[21] Collaborators GS (2017). Measuring progress and projecting attainment on the basis of past trends of the health-related sustainable development goals in 188 countries: an analysis from the global burden of disease study 2016. Lancet.

[22] Mortality GBD (2016). Causes of death C. Global, regional, and national life expectancy, all-cause mortality, and cause-specific mortality for 249 causes of death, 1980-2015: a systematic analysis for the global burden of disease study 2015. Lancet.

[23] Devleesschauwer B, Havelaar AH, Maertens de Noordhout C, Haagsma JA, Praet N, Dorny P, et al. Calculating disability-adjusted life years to quantify burden of disease. International journal of public health. 2014; 10.1007/s00038-014-0552-z 24752429

[24] Devleesschauwer B, Havelaar AH, Maertens de Noordhout C, Haagsma JA, Praet N, Dorny P, et al. DALY calculation in practice: a stepwise approach. International journal of public health. 2014; 10.1007/s00038-014-0553-y 24748107

[25] Boerma T, Victora C, Abouzahr C. Monitoring country progress and achievements by making global predictions: is the tail wagging the dog? Lancet. 2018; 10.1016/S0140-6736(18)30586-5 29661480

[26] Murray CJ, Richards MA, Newton JN, Fenton KA, Anderson HR, Atkinson C (2013). UK health performance: findings of the global burden of disease study 2010. Lancet.

[27] Bossuyt N, Van Casteren V (2007). Epidemiology of suicide and suicide attempts in Belgium: results from the sentinel network of general practitioners. International journal of public health..

[28] Vancayseele N, De Jaegere E, Portzky G, van Heeringe K. Time trends and geographical distribution of attempted suicide in Flanders (Belgium). 2010. Available on: https://www.eenheidzelfmoordonderzoek.be/pdf/14042014-154337-poster2.pdf.

[29] Van Oyen H, Berger N, Nusselder W, Charafeddine R, Jagger C, Cambois E (2014). The effect of smoking on the duration of life with and without disability, Belgium 1997-2011. BMC Public Health.

[30] Yokota RT, Nusselder WJ, Robine JM, Tafforeau J, Deboosere P, Van Oyen H (2016). Contribution of chronic conditions to the disability burden across smoking categories in middle-aged adults, Belgium. PLoS One.

[31] Newton JN, Briggs AD, Murray CJ, Dicker D, Foreman KJ, Wang H (2015). Changes in health in England, with analysis by English regions and areas of deprivation, 1990-2013: a systematic analysis for the global burden of disease study 2013. Lancet.

