# Supplementary material for: Changes in health in Belgium, 1990–2016: a benchmarking analysis based on the global burden of disease 2016 study
Source: BMC Public Health. 2018 Jun 20;18:775. doi: 10.1186/s12889-018-5708-y (PMC6011511; doi:10.1186/s12889-018-5708-y)
Supplement: Supplementary file 1 — Includes six additional figures and additional results on risk factors linked with DALYs in Belgium in 2016. Figure S9 in the Additional file 1 represents the top 15 causes of age-standardized (AS) deaths per 100,000 by sex in 2016 in Belgium. Fig. S10 represents the AS YLDs per 100,000 in EU15 in 1990 and 2016. Fig. S11 represents the ranking (descending) and contribution of health states by AS YLDs per 100,000 in 1990 and 2016 in males in Belgium. Fig. S12 represents the ranking (descending) and contribution of health states by AS YLDs per 100,000 in 1990 and 2016 in females in Belgium. Finally, we presented additional results on risk factors linked with DALYs in Belgium in 2016 and Fig. S13 and S14 represent selected disorders attributable to risk factors linked with DALYs in males and females in 2016 in Belgium. (DOCX 474 kb) [file 12889_2018_5708_MOESM1_ESM.docx]

**Additional file 1 – “Changes in health in Belgium, 1990-2016: a benchmarking analysis based on the Global Burden of Disease 2016 study”.**

**Figure 9: Top 15 causes of age-standardized deaths per 100,000 by sex, 2016, Belgium.**


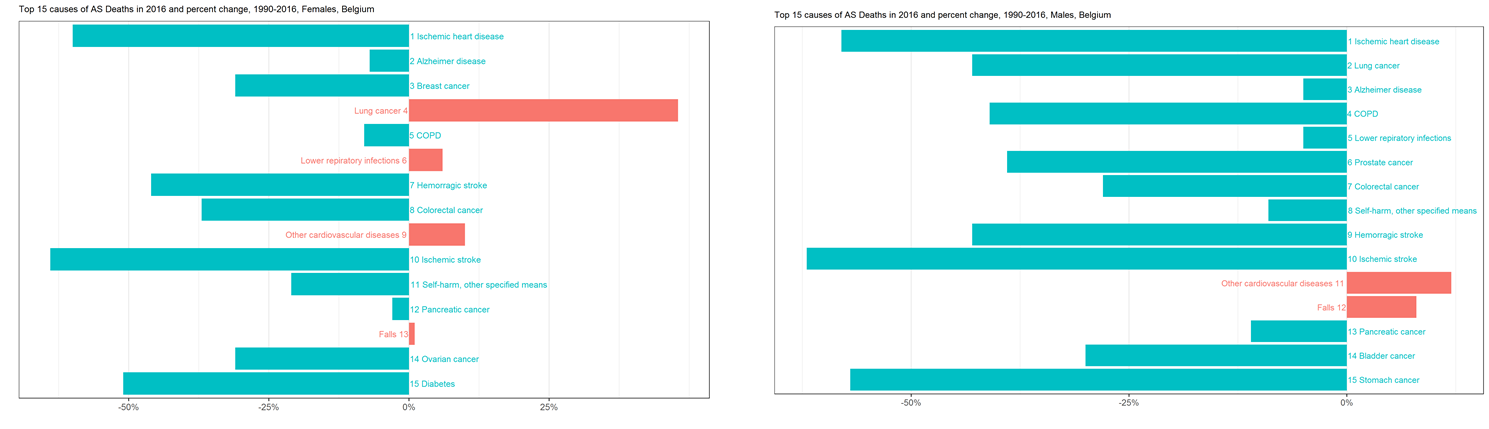


COPD = Chronic Obstructive Pulmonary Disease. Other cardiovascular diseases = cardiovascular and circulatory diseases different than rheumatic heart disease, ischemic heart disease, cerebrovascular disease, hypertensive heart disease cardiomyopathy and myocarditis, atrial fibrillation and flutter, aortic aneurysm, peripheral vascular disease and endocarditis.

**Figure 10: Age-standardized Years Lived with Disability (YLDs) per 100,000, EU15.**

**
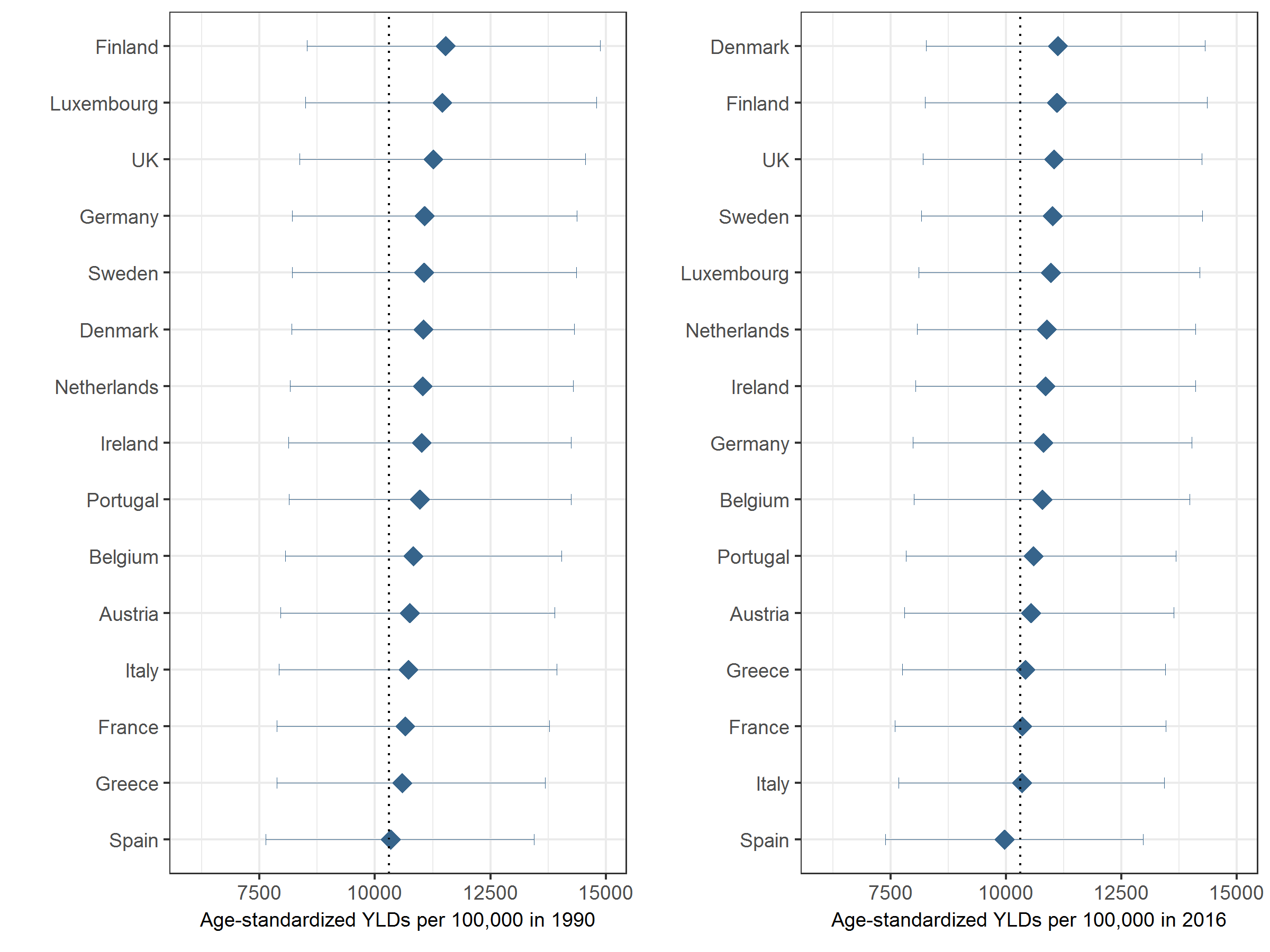
**

Diamonds represent total YLDs per 100,000 per EU15 country, horizontal line equals 95% UI YLDs, dashed line equals EU15 mean

**Figure 11: Ranking (descending) and contribution of health states by age-standardized (AS) Years Lived with Disability (YLDs) per 100,000, 1990 and 2016, males, Belgium.**

**Figure 12: Ranking (descending) and contribution of health states by age-standardized (AS) Years Lived with Disability (YLDs) per 100,000, 1990 and 2016, females, Belgium.**

**Additional results on risk factors linked with DALYs, Belgium, 2016.**

Risk factors linked with top three health states that caused most of the DALYs in males and females in Belgium in 2016, i.e. in males, ischemic heart disease (IHD), low back and neck pain (LBP), tracheal-bronchus and lung (TBL) cancer and in females, LBP, IHD and major depressive disorder.

In males, behavioral and metabolic risk factors caused most of the DALYs linked with IHD (51.6%), although tracheal, bronchus and lung cancer were largely caused by behavioral risk factors (44.0%). Low-back and neck pain was mainly not attributable to GBD risk factors (79.9%) (Figure 5).

In females, behavioral and metabolic risk factors caused most of the DALYs linked with IHD (50.5%), although major depressive disorder and low-back and neck pain was mainly not attributable to the considered risk factors (depressive disorder: 86.5% - low back and neck pain: 84.7%) (Figure 6).

**Figure 13: Selected disorders attributable to risk factors, Disability-Adjusted Life Years, males, 2016, Belgium**


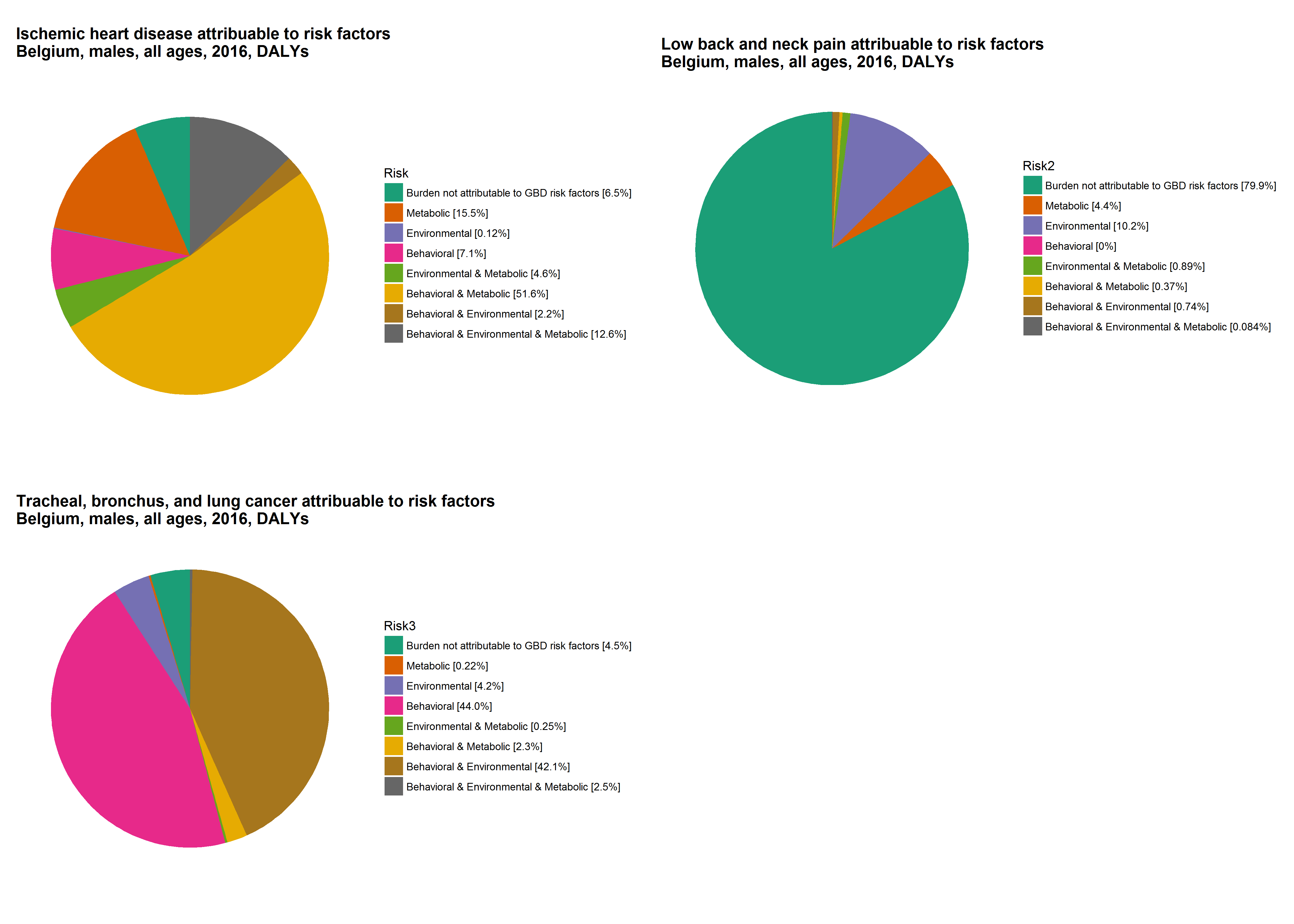


**Figure 14: Selected disorders attributable to risk factors, Disability-Adjusted Life Years, females, 2016, Belgium**

**
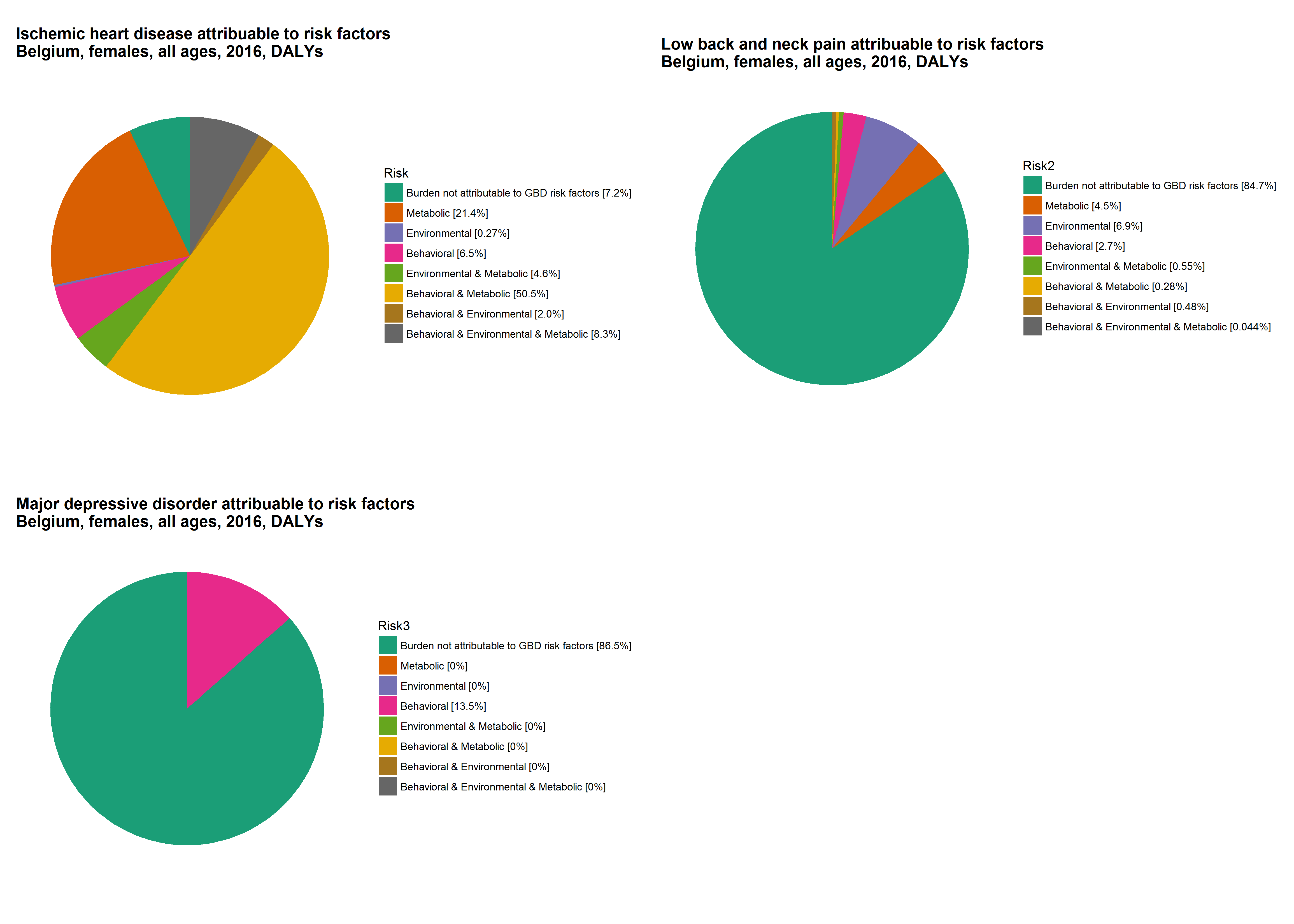
**
